# Mycobacterium abscessus Tetracycline-Modifying Monooxygenase *MAB_1496c* Appears Not to Be Sufficient to Cause Resistance to Tetracycline When Expressed in Mycobacterium smegmatis

**DOI:** 10.1128/spectrum.02346-22

**Published:** 2022-07-27

**Authors:** Noga Naor, Erez Zarbib, Daniel Barkan

**Affiliations:** a Koret School of Veterinary Medicine, The Robert H. Smith Faculty of Agriculture, Food and Environment, The Hebrew University of Jerusalem, Rehovot, Israel; b Department of Life Sciences, Ben-Gurion University of the Negev, Beer-Sheva, Israel; Johns Hopkins University School of Medicine

**Keywords:** tetracycline, mycobacteria, antibiotic resistance, *Mycobacterium*, drug resistance mechanisms, *Mycobacterium abscessus*

## LETTER

Recently, a gene in Mycobacterium abscessus (*MAB_1496c*) coding for a FAD-binding monooxygenase protein was described (1, 2). It was shown that the protein bears resemblance to tetracycline destructases (TetX) from other bacteria (3), that deletion of the gene rendered M. abscessus substantially more sensitive to tetracycline, and that this resistance was unrelated to the *whiB7* pathway (4, 5). The protein was also shown to modify tetracycline *in vitro* by monooxygenation and to be inhibited by a low concentration of the tetracycline analog anhydrotetracycline (ATc) (1, 6). These findings suggested that the protein coded by *MAB_1496c* can induce tetracycline resistance in those mycobacteria that are naturally more sensitive to tetracycline than M. abscessus, such as M. smegmatis and M. tuberculosis (7, 8). This could be used for research and biotechnology purposes, such as *in vitro* positive selection (9, 10).

**FIG 1:**
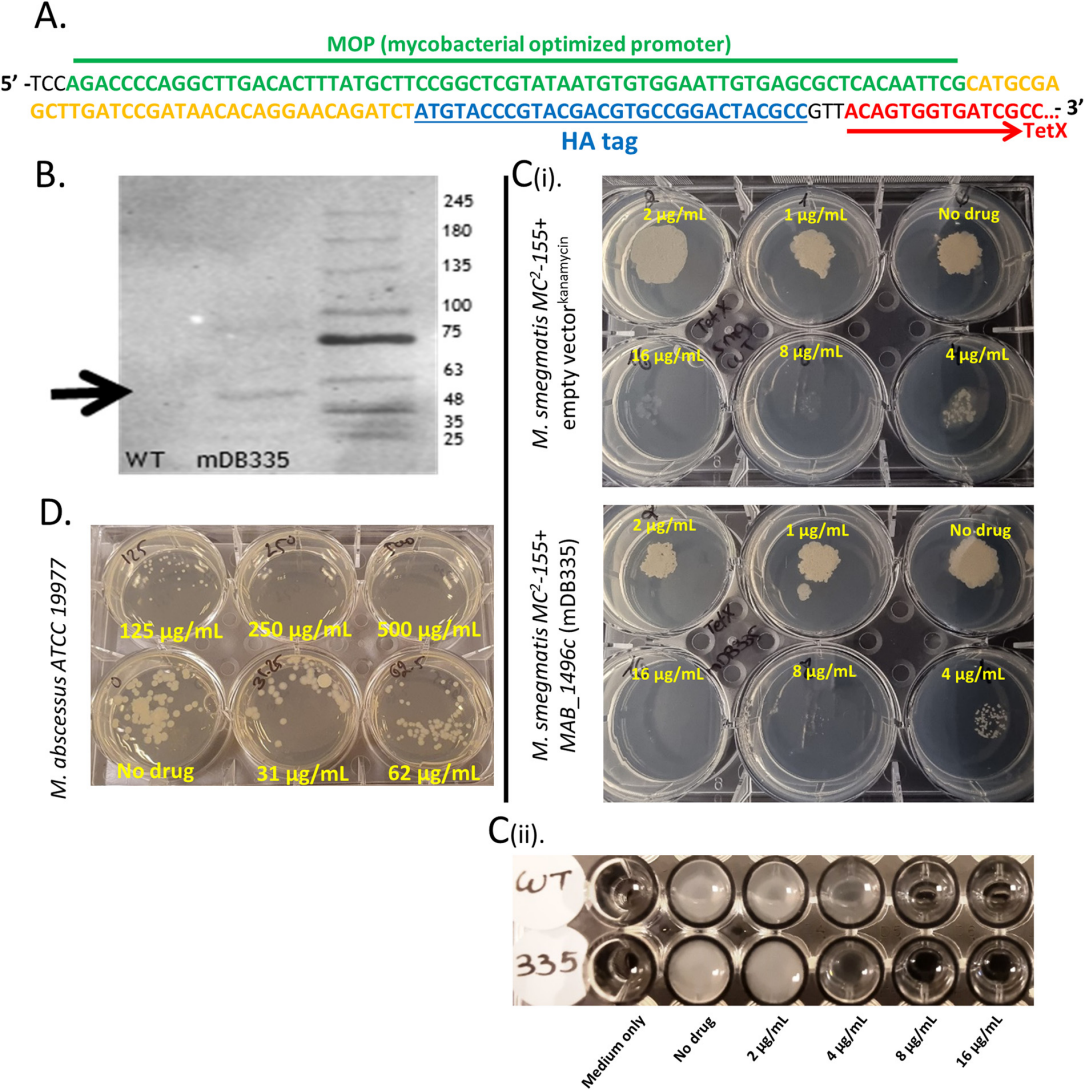
(A) Genetic sequences of the MOP promoter (green) and untranslated region (UTR) (yellow) driving the expression of an HA-tagged (blue) TetX protein (red). (B) Western blot demonstrating the full-length (53.2-kDa) HA-TetX protein in mDB335 in comparison to WT bacteria. (Ci) Approximately 250 CFU of M. smegmatis^empty kana vector^ (top) or mDB335 (bottom) was plated onto 7H10 agar plates with increasing concentrations of tetracycline. (Cii) WT M. smegmatis and M. smegmatis mDB335 were grown in 7H9/glycerol broth with the designated concentrations of tetracycline. (D) M. abscessus ATCC 19977 was plated onto 7H10 agar plates with the designated concentrations of tetracycline.

We therefore opted to examine whether the recombinant expression of *MAB_1496c* (MabTetX protein) in M. smegmatis would substantially increase the bacterium’s MIC to tetracycline, to the point where it could be used for *in vitro* selection. We cloned *MAB_1496c* into the blunt HpaI site in pDB32—a multicopy, kanamycin-selected, episomal vector (11). The gene was PCR amplified from M. abscessus genomic DNA (gDNA) using the following primers: forward, 5′-ACAGTGGTGATCGCCGGGGCCGGCC-3′; reverse, 5′-CATCTAGACAACACGGGCGAGATA-3′. Cloning into the HpaI site of this vector places the gene in frame with a hemagglutinin (HA) tag at the N terminus and under a constitutive mycobacterial optimized promoter (MOP). As the native promoter of *MAB_1496c* is regulated by *MAB_1497c* (5), a *tetR*-like transcription regulator (and is induced by tetracycline), placing the recombinant construct under a constitutive promoter avoids problems resulting from regulation of expression. The genetic structure of the construct is shown in Fig. 1A for clarity. Correct in-frame cloning with the HA tag was confirmed by Sanger sequencing, and the plasmid (pDB451) was electroporated into wild-type (WT) M. smegmatis MC^2^ 155 to produce mDB335. The expression of the full-length MabTetX protein was verified by Western blotting using anti-HA antibodies (Abcam), showing a protein at the expected size of 53.2 kDa (Fig. 1B). However, when we tested the TetX-expressing mutants for tetracycline sensitivity, we found no difference between their MICs and that of WT M. smegmatis with an empty vector on either 7H10 agar plates (Fig. 1Ci) or in 7H9/glycerol broth (Fig. 1Cii). The MIC also remained well below that of WT M. abscessus (Fig. 1D). Of note, when we examined the MIC to tetracycline in the E. coli bacteria used for the cloning (DH5-α), we again found bacteria with the plasmids to have the same MIC to tetracycline as “WT” E. coli bacteria, with the empty control vectors (data not shown).

To summarize, despite the convincing results of the carefully performed experiments reported in the original description (1), it appears that the full picture is more complex, and expression of the MAB_1496c protein is, by itself, not sufficient to induce tetracycline resistance in M. smegmatis. This could be due to a requirement for another, yet unidentified cofactor, low activity of the protein in the M. smegmatis context, retained antimicrobial activity of the oxygenated tetracycline product against M. smegmatis, or misfolding of the protein in M. smegmatis, resulting in a non- or poorly functional, albeit full-size, protein.
